# Cryoablation in the treatment of early breast cancer: a comprehensive analysis

**DOI:** 10.3389/fonc.2025.1469684

**Published:** 2025-05-20

**Authors:** Yanli Xing, Hongmei Li, Ting Yuan, Xiaojiao Zou, Bing Liang, Yangyang Ma, Lizhi Niu

**Affiliations:** ^1^ Department of Oncology, Guangzhou Fuda Cancer Hospital, Guangzhou, China; ^2^ Department of Surgery and Anesthesia, Guangzhou Fuda Cancer Hospital, Guangzhou, China; ^3^ Central Laboratory, Guangzhou Fuda Cancer Hospital, Guangzhou, China

**Keywords:** cryoablation, early breast cancer, local treatment, efficacy assessment, clinical applications

## Abstract

Cryoablation has emerged as a promising local treatment technique for early breast cancer, garnering significant interest in recent years. This review delves into the fundamental principles of cryoablation, its clinical applications, efficacy assessments, and comparisons with traditional treatment modalities. We explore the potential advantages and challenges associated with incorporating cryoablation into the management of early breast cancer. Furthermore, we analyze current research developments and future directions in this field, aiming to provide valuable insights for clinical practice and enhance patient care in breast cancer management.

## 1 Introduction

Breast cancer remains one of the most prevalent malignancies globally, with a significant impact on women’s health. The rising incidence rates have prompted a critical reevaluation of treatment strategies, particularly for early-stage breast cancer, which presents unique challenges and opportunities for innovative therapies (1–4). Traditional treatment options such as surgery, radiation therapy, and chemotherapy have been the cornerstone of management. However, the focus is shifting towards minimizing treatment-related morbidity while maximizing patient quality of life. In this context, cryoablation has emerged as a promising minimally invasive alternative to conventional surgical approaches, offering potential benefits in terms of safety, efficacy, and patient satisfaction (5–7).

The treatment landscape for early-stage breast cancer has evolved, emphasizing the need for less aggressive interventions without compromising oncological outcomes. Surgical options, such as lumpectomy and mastectomy, while effective, often entail significant physical and emotional burdens on patients. Cryoablation has emerged as a promising minimally invasive technique for the treatment of various cancers, including early-stage breast cancer (8–14). This technique involves the application of extreme cold to destroy cancerous tissues, offering potential advantages over conventional treatments, such as reduced recovery times and fewer complications (15–17). Early clinical trials have demonstrated that cryoablation can achieve comparable local control rates to surgical excision, particularly in tumors less than 2 cm in size, with low recurrence rates reported (18, 19). Moreover, cryoablation can be performed in an outpatient setting under local anesthesia, thereby reducing recovery times and enhancing patient comfort (20).The rise of cryoablation in oncology is driven by its ability to precisely target tumor cells while sparing surrounding healthy tissues, thus minimizing collateral damage and enhancing patient quality of life (21–24).

Despite the promising results, the integration of cryoablation into clinical practice requires careful consideration of patient selection criteria and comprehensive management protocols. The current literature highlights a need for standardized guidelines to optimize patient outcomes and ensure that cryoablation is utilized effectively within the broader spectrum of breast cancer treatment (25, 26). This review aims to clarify cryoablation’s role in early-stage breast cancer management, highlighting benefits and limitations to aid clinicians and researchers in optimizing treatment protocols.

## 2 Cryoablation for early breast cancer

### 2.1 Principles and equipment of cryoablation technology

#### 2.1.1 Basic principles of cryoablation

Cryoablation is a minimally invasive technique that employs extreme cold to destroy cancer cells (14, 27, 28). The fundamental principle involves the application of freezing temperatures to induce cellular injury and death (29, 30). This process is typically achieved using cryoprobes that deliver liquid nitrogen or argon gas to the target tissue, creating an “iceball” that encompasses the tumor (31). The rapid freezing and slow thawing cycles cause ice crystals to form within the cells, leading to cellular dehydration, membrane rupture, and ultimately, cell death through necrosis and apoptosis (32, 33). Additionally, cryoablation induces vascular damage, resulting in ischemia and further contributing to tumor destruction (33–35). The technique’s efficacy is enhanced by the immune response triggered by the release of tumor antigens during the cryoablation process, potentially aiding in the systemic eradication of cancer cells (16, 36–40).

The effectiveness of cryoablation is particularly notable in the treatment of early-stage breast cancer, where it has been demonstrated to achieve high rates of tumor ablation and low recurrence rates (41). Studies indicate that cryoablation can be performed safely in an outpatient setting, often under local anesthesia, which minimizes patient discomfort and recovery time compared to traditional surgical approaches (18, 25, 42). The technique is gaining traction as a viable alternative to surgical resection, especially for patients with small, low-risk tumors who may not tolerate more invasive procedures (43).

#### 2.1.2 Mechanism of cryoablation

**Figure 1:**
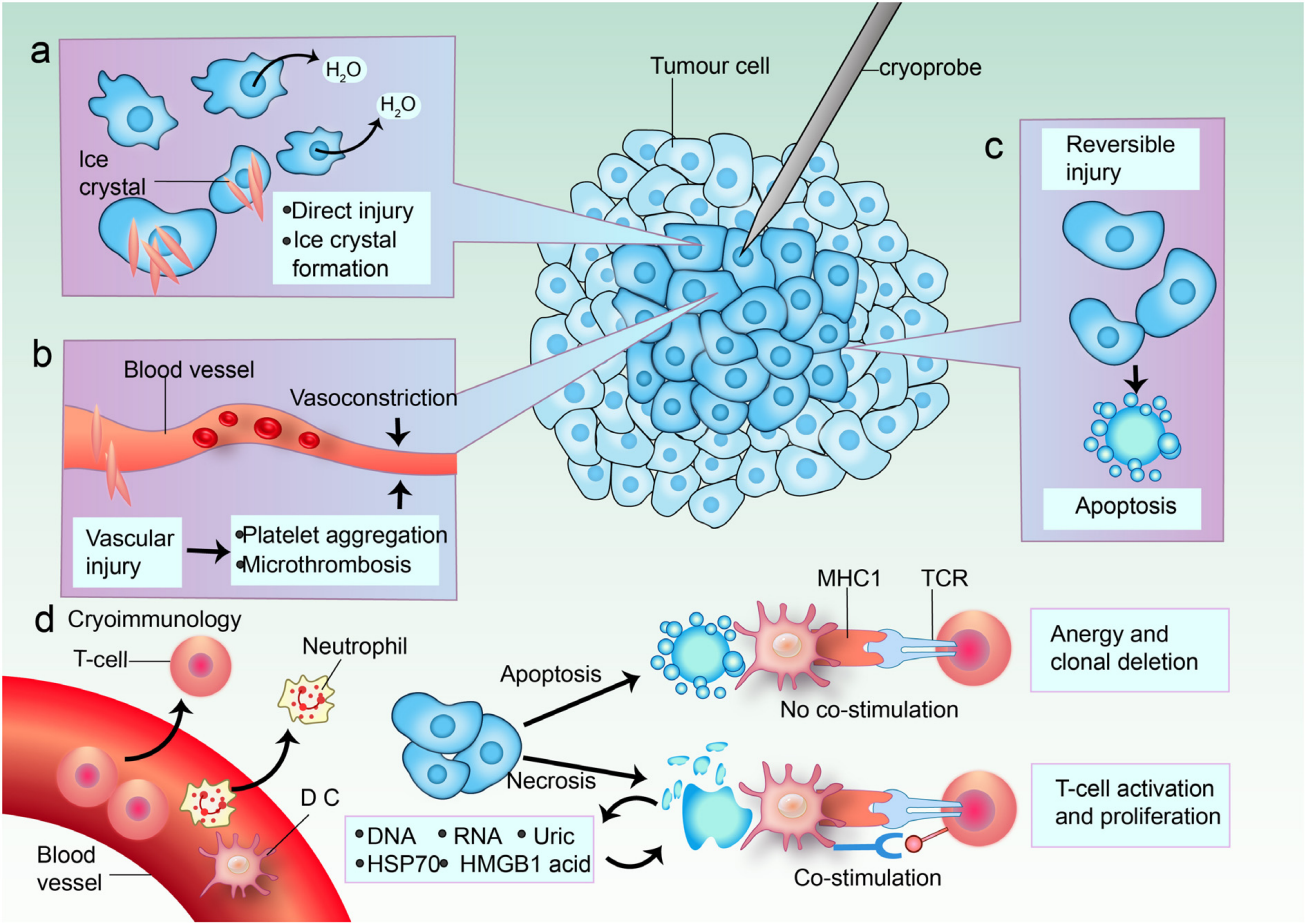
Mechanisms of cell death in cryoablation. **(a)** In the center of the cryoablative lesion lies a sharply delineated area of frozen necrosis where direct injury takes place. Here, the temperature rapidly drops below -40°C, causing ice to form from the extracellular space inward. This leads to a hypertonic extracellular environment and osmotic cell shrinkage due to fluid shifting out of the cell. The formation of ice crystals increases direct damage. **(b)** Cold-induced vascular injury causes harm to endothelial cells and cell junctions, resulting in platelet aggregation and microthrombosis. Vasoconstriction occurs in response to cooling temperatures. Freezing also leads to a hyperemic response and increased vascular permeability. The consequence is ischemia, causing further coagulative necrosis. **(c)** Apoptosis occurs in a peripheral zone of sublethal cold temperatures, and this is likely induced by reversible damage. **(d)** Blood vessels supply immune cell infiltrates. Both enhanced and reduced anti-tumor immunity can be triggered by cryoablation; immunomodulation might depend on the predominant mode of cell death. Some tumor cells undergo apoptosis. When antigen-presenting cells (APCs) such as dendritic cells (DCs) and macrophages phagocytose tumor cells after apoptosis without danger signals, the tumor antigens are presented on major histocompatibility complex (MHC) class I molecules without co-stimulation of T cells. The dying cells can even secrete immunosuppressive cytokines, such as interleukin-10 (IL-10) and transforming growth factor-β (TGFβ). This induces anergy and clonal deletion. Other tumor cells are necrotic, and they release their extracellular contents: DNA, RNA, heat shock protein 70 (HSP70), uric acid, and high mobility group protein B1 (HMGB1). Pro-inflammatory cytokines induce DCs to take up more antigens and express danger signals via co-stimulatory molecules that are necessary to prime nearby T cells. TCR, T cell receptor.

The mechanism of cryoablation involves several key processes that contribute to its effectiveness in tumor destruction. Initially, the application of extreme cold leads to the formation of ice crystals within the tumor cells, which disrupts cellular membranes and organelles, ultimately causing cell death through necrosis. This process is facilitated by the rapid cooling of the tissue, which can reach temperatures as low as -40°C, effectively inducing irreversible damage to the cellular structure (19, 44–46). Moreover, cryoablation has been found to induce an inflammatory response that can enhance the immune system’s ability to recognize and attack tumor cells (47–49). The necrotic tissue releases tumor-specific antigens into the systemic circulation, potentially activating both innate and adaptive immune responses (16, 40, 50). This immunological effect is particularly relevant in the context of combining cryoablation with immunotherapy, as it may enhance the overall therapeutic efficacy against the tumor (16, 51, 52). Additionally, cryoablation precisely targets tumors while protecting healthy tissue, minimizing damage and preserving adjacent structures, aided by imaging guidance like ultrasound or MRI for real-time monitoring (53) (**Figure 1**).

#### 2.1.3 Cryoablation equipment and techniques

Cryoablation equipment is characterized by its precision, safety, and efficacy. These systems are designed to deliver controlled freezing cycles, ensuring complete ablation of the target tissue while minimizing damage to adjacent structures, including real-time imaging capabilities, such as ultrasound, CT, or MRI guidance, which allow for accurate placement of cryoprobes and monitoring of the ablation process (54). Additionally, these devices offer adjustable cryoprobe sizes and configurations, enabling customization based on tumor size and location (55). Safety mechanisms, such as temperature sensors and feedback systems, are integrated to prevent over-freezing and reduce the risk of complications (56).

### 2.2 Clinical application of cryoablation in early breast cancer

#### 2.2.1 Definition and treatment principles of early breast cancer

Early breast cancer is typically defined as cancer that is confined to the breast or has only spread to nearby lymph nodes but not to distant parts of the body (57–59). The primary treatment principles for early breast cancer include local control of the tumor and prevention of systemic recurrence (3, 60–62). Local control is often achieved through surgery, which may be followed by radiation therapy, while systemic recurrence is managed with adjuvant therapies such as chemotherapy, hormone therapy, and targeted therapy (63). The goal is to eliminate cancer cells and reduce recurrence risk, improving survival and quality of life (64).

#### 2.2.2 Indications for cryoablation in early breast cancer treatment

Cryoablation is indicated for early breast cancer in patients with small, localized tumors under 1.5 cm, especially low-grade, hormone receptor-positive ones (43, 65, 66). Patients unsuitable for surgery may benefit from this treatment, while contraindications include large tumors, multifocal disease, and significant comorbidities (25). Moreover, patients with certain types of breast cancer, such as triple-negative breast cancer, may not be ideal candidates for cryoablation due to the aggressive nature of the disease and the potential for higher recurrence rates. It is essential for healthcare providers to conduct a thorough assessment of each patient’s clinical profile before recommending cryoablation as a treatment option.

#### 2.2.3 Clinical trials and research findings

Cryoablation has emerged as a minimally invasive therapeutic modality for both benign and malignant breast pathologies (26, 67). For fibroadenoma management, this technique demonstrates sustained efficacy with longitudinal studies reporting >85% volumetric reduction and excellent post-procedural cosmesis (68, 69). The clinical application has expanded to invasive carcinomas, achieving histological ablation success rates of 76-100% in neoplasms ≤2 cm when utilizing modern cryogenic systems (27, 70) (**Table 1**).

**Table 1:** Summary of cryoablation on early breast cancer clinical studies.

Study (Year)	Design	Population/Key Criteria	Intervention	Key Outcomes	Ref.
Sabel et al. (2004)	Prospective cohort study	27 patients Invasive carcinoma ≤1.5 cm <25% DCIS	US-guided cryoablation (Visica system, argon gas, double freeze-thaw)	100% ablation in tumors ≤1.0 cm; 100% success in 1.0–1.5 cm tumors with <25% DCIS	(66)
Roubidoux et al. (2004)	Case series	9 patients Invasive carcinoma ≤1.8 cm	Cryoablation + surgical resection	100% ablation in tumors ≤1.7 cm; Residual disease in tumors >1.7 cm or multifocal DCIS	(71)
ACOSOG Z1072 (2016)	Phase II clinical trial	86 patients Unifocal IDC ≤2 cm <25% DCIS	Cryoablation (Visica2 system) + resection	75.9% complete ablation; 92% success in non-multifocal tumors <2 cm; 100% in ≤1 cm	(70)
Roca Navarro et al. (2023)	Prospective cohort study	20 patients ER+/HER2- IDC ≤2 cm	Triple-phase cryoablation (ICEfX system)	95% complete response; 1 case of <1 mm residual disease	(72)
ICE3 Trial (2014–)	Prospective interventional trial	194 patients Age ≥60 years, Grade ≤2 tumors ≤1.5 cm	Liquid nitrogen cryoablation (ProSense system)	2.06% 3-year recurrence; 96.4% 5-year recurrence-free survival; 95% patient satisfaction	(27)

Early pivotal work by Sabel et al. (66) utilized an argon gas-based cryoablation system (Visica) delivering probe temperatures of -160°C via a double freeze-thaw protocol. In 27 patients with invasive carcinomas, 100% ablation success was achieved for tumors ≤1.0 cm (**Table 1**). For lesions 1.0–1.5 cm, equivalent efficacy required <25% ductal carcinoma *in situ* (DCIS) on core biopsy. Technical refinements during the trial, including probe insulation optimization (2.7 mm diameter probe with distal 4 cm cooling), highlighted the critical interplay between device design and tumor size limitations. Concurrently, Roubidoux et al. (71) reported complete tumor eradication in 9 patients with invasive carcinomas ≤1.7 cm, while residual disease was observed in larger tumors (1.8 cm) or those with undetected multifocal DCIS, reinforcing the importance of preoperative imaging-pathology correlation (**Table 1**).

The ACOSOG Z1072 phase II trial (70) prospectively evaluated cryoablation in 86 patients with unifocal invasive ductal carcinoma (IDC) ≤2 cm and <25% DCIS. Employing the Visica2 system with pre- and post-procedural MRI, pathologic analysis revealed: 75.9% complete ablation across all cases, 92% success in non-multifocal tumors <2 cm, 100% efficacy in tumors ≤1.0 cm. These findings underscored multifocality as a key predictor of treatment failure.

Recent advancements by Roca Navarro et al. (72) integrated magnetic seed localization (Sentimag) with triple-phase cryoablation (ICEfX system, 14-G/17-G probes) under continuous ultrasound guidance (**Table 1**). In 20 patients with ER+/HER2- IDC <2 cm, histologic analysis post-excision (6–49 days) demonstrated 95% complete ablation (19/20 cases), with a single case showing <1 mm residual invasive disease. Critical Exclusion Criteria: Invasive lobular carcinoma remains contraindicated due to its multicentric growth pattern and poor correlation between imaging findings and pathologic extent (73).

The therapeutic paradigm for early-stage breast cancer is being redefined by three landmark clinical trials evaluating cryoablation as definitive treatment. The ICE3 trial (NCT02200705), initiated in 2014, enrolled 194 elderly patients (mean age 75 years) with ultrasound-visible unifocal IDC ≤1.5 cm (ER/PR+, HER2-, Nottingham grade ≤2) (27) (**Table 1**). Utilizing the ProSense liquid nitrogen system (-170°C), this protocol achieved 35–40 mm ice balls through stab-incision placement with dual freeze-thaw cycles. Three-year interim analysis revealed 2.06% ipsilateral recurrence (4/194), comparable to surgical outcomes, with 20.8% experiencing minor adverse events (ecchymosis, transient cryodermatitis) (27) (**Table 1**). Extended 5-year follow-up demonstrated 96.4% local control (7 recurrences) and universal satisfaction with cosmetic outcomes, despite three undetermined breast cancer-related mortalities (74).

These trials collectively suggest cryoablation achieves 96-99% 5-year local control in carefully selected, imaging-concordant IDC. However, critical knowledge gaps persist regarding optimal patient selection biomarkers, DCIS component thresholds, and long-term survival equivalence to surgical resection. The integration of genomic profiling (COOL-IT) and artificial intelligence-driven thermal monitoring represents the next frontier in ablative precision oncology.

### 2.3 Evaluation of the efficacy of cryoablation

#### 2.3.1 Tumor control rate

The tumor control rate is a critical metric for evaluating the efficacy of cryoablation. It reflects the ability of the procedure to effectively manage tumor growth and prevent recurrence. Various studies have reported favorable tumor control rates across different types of cancers treated with cryoablation. For example, in patients with renal cell carcinoma, cryoablation has shown a local recurrence-free survival rate of approximately 88.1% at one year, with significant long-term control observed in many cases (75). In breast cancer, cryoablation has demonstrated a complete ablation rate of 90%, indicating its effectiveness in achieving tumor control without the need for extensive surgical procedures (18). Additionally, a meta-analysis comparing cryoablation with radiofrequency ablation for non-small cell lung cancer revealed that cryoablation had superior disease-free survival rates, further supporting its efficacy as a treatment option (76). These findings collectively highlight that cryoablation can achieve high tumor control rates, making it a valuable option in the oncological treatment landscape, particularly for patients with localized tumors.

#### 2.3.2 Analysis of postoperative complications and safety

The safety profile of cryoablation is a significant aspect of its overall efficacy evaluation. While the technique is generally associated with fewer complications compared to traditional surgical approaches, it is essential to analyze the types and rates of postoperative complications that may arise. Studies indicate that cryoablation is associated with a lower incidence of severe complications, particularly in comparison to more invasive surgical procedures (77–79). Additionally, a study on cryoablation for breast lesions reported no major complications in patients, emphasizing the procedure’s safety (80). Minor complications like pain or swelling can occur, but the overall risk is low, making cryoablation an appealing option for patients wanting less invasive treatments. Furthermore, advanced imaging in cryoablation improves precision, reducing complications from misplacement or inadequate tumor treatment (53). Cryoablation’s safety profile, with low complications and good outcomes, confirms its viability in modern oncology.

#### 2.3.3 Imaging techniques for post-cryoablation efficacy assessment

Ultrasound: Ultrasound has become the most commonly used method for assessing treatment efficacy after cryoablation due to its real-time capabilities, ease of operation, and lack of radiation exposure. Post-cryoablation ultrasound evaluates treatment efficacy by detecting changes in low echo areas and the absence of Doppler blood flow signals. This is particularly helpful for accurately locating tumor boundaries and assessing immediate treatment effects (18, 81, 82).

Magnetic Resonance Imaging (MRI): MRI provides clear images of breast tumors and surrounding structures, making it widely used for assessing the necrosis extent in the ablation area and the presence of residual tumor activity. However, the MRI-guided cryoablation process is time-consuming and requires expensive equipment, posing significant challenges for its widespread clinical adoption (83, 84).

Computed Tomography (CT): CT, with its high spatial resolution, clearly shows breast tumor lesions and surrounding structures, making it especially suitable for deeper or more complex lesions (23, 85). However, CT guidance involves radioactive radiation, which limits its conventional application.

### 2.4 Comparison of cryoablation with traditional treatment methods

Cryoablation is a minimally invasive treatment with advantages over surgery and radiation, offering a compelling option for tumors while reducing risks. The technique involves the application of extreme cold to destroy cancerous cells, resulting in less damage to surrounding healthy tissue, shorter recovery times, and improved cosmetic outcomes. Compared to surgical resection, which often requires significant recovery time and carries the risks of major surgery, cryoablation is associated with lower complication rates and can be performed on an outpatient basis (53, 86, 87). This suggests that cryoablation may be a suitable alternative for patients who are not candidates for surgery due to comorbidities or personal preferences.

#### 2.4.1 Comparison with surgical resection

When comparing cryoablation to surgical resection, it is essential to consider both the efficacy and safety profiles of the two approaches. Surgical resection remains the gold standard for many solid tumors, particularly when complete removal of the tumor is feasible. However, the invasiveness of surgical procedures can lead to longer hospital stays, increased postoperative pain, and a higher risk of complications (88). In contrast, cryoablation is associated with shorter recovery times and less postoperative discomfort. Studies have reported that patients undergoing cryoablation experience significantly lower rates of treatment-emergent adverse events compared to those undergoing surgical resection (89, 90). Moreover, cryoablation can be repeated for tumor recurrence, offering flexibility, while preserving healthy tissue and organ function, making it appealing for localized tumors despite surgical resection being more definitive (91, 92). Ultimately, the choice between cryoablation and surgical resection should be individualized based on tumor characteristics, patient preferences, and overall health status.

#### 2.4.2 Combined application with radiotherapy

The combination of cryoablation with radiotherapy represents a promising strategy for enhancing treatment efficacy in various malignancies. While cryoablation effectively targets and destroys tumor cells through extreme cold, radiotherapy complements this by delivering ionizing radiation to eliminate residual cancer cells and reduce the risk of local recurrence (22, 93, 94). This synergistic approach has shown potential in improving overall survival rates and disease-free survival (94). The integration of cryoablation with radiotherapy may also exploit the abscopal effect, where localized treatment leads to systemic immune responses that can target distant metastases (95). For example, in patients with metastatic dedifferentiated liposarcoma, cryoablation has been observed to induce immune-mediated regression of untreated metastases, suggesting that combining local therapies with immunotherapy could enhance therapeutic outcomes (96). Furthermore, using cryoablation as a bridging therapy prior to immunotherapy, such as CAR-T cell therapy, has shown promising results in reducing tumor burden and improving response rates (97). Overall, the combination of cryoablation with radiotherapy offers a multifaceted approach to cancer treatment, potentially maximizing therapeutic efficacy while minimizing treatment-related morbidity.

### 2.5 Future directions of cryoablation

Cryoablation, a minimally invasive technique that employs extreme cold to destroy abnormal tissue, has shown promising results in various clinical applications. As the field continues to evolve, the future of cryoablation appears to be shaped by significant technological advancements and innovations. Recent developments in imaging techniques, such as MRI and ultrasound, have enhanced the precision of cryoablation procedures, allowing for better targeting of lesions and improved patient outcomes (98). Moreover, the integration of robotic systems and automated devices is expected to streamline the procedure, reduce operator variability, and enhance safety during ablation. Innovations in cryoablation devices, including multi-needle systems that allow for simultaneous treatment of multiple sites, are also on the horizon (99). Furthermore, the incorporation of nanotechnology in cryoablation could improve the delivery of cryogenic agents and enhance the effectiveness of the treatment by modulating the immune response post-ablation (100, 101). Overall, the technological progress in cryoablation is likely to expand its applications across various malignancies and benign conditions, leading to more effective and personalized treatment options.

#### 2.5.1 Technological advances and innovations

The cryoablation technology is advancing rapidly, with innovations improving efficacy and safety, particularly in imaging modalities like real-time MRI and ultrasound, which enhance tissue localization and reduce damage to healthy structures (53). Advancements in cryoablation devices, like temperature-sensitive materials and better cryogenic agents, will optimize freezing for more effective ablation (102). Furthermore, the integration of AI and machine learning in cryoablation systems promises to automate procedures and enhance decision-making in treatment (103, 104). These innovations improve cryoablation’s precision and safety while enabling new tumor treatments.

#### 2.5.2 Clinical applications: prospects and challenges

The clinical application of cryoablation is expanding, with promising prospects in treating a variety of conditions (99). Cryoablation’s effectiveness as a minimally invasive alternative is likely to boost its clinical adoption. However, several challenges remain. One of the primary obstacles is the need for standardized protocols and guidelines to ensure consistent outcomes across different institutions and practitioners (105). Additionally, while cryoablation is associated with lower morbidity compared to surgical resection, concerns regarding the long-term efficacy and potential for recurrence still need to be addressed through ongoing research and clinical trials (103). Furthermore, the integration of cryoablation into multimodal treatment plans, particularly in combination with immunotherapy or targeted therapies, presents both opportunities and challenges that require careful consideration and investigation (16, 37, 105–107). Overall, while the future of cryoablation is promising, addressing these challenges will be crucial for its successful implementation in clinical settings.

## 3 Conclusion

Cryoablation for early-stage breast cancer shows promise but has limitations; it offers minimally invasive options to reduce morbidity while ensuring effective outcomes, necessitating comparison with traditional surgeries. Balancing diverse findings on cryoablation is challenging; some studies show good tumor control and cosmetic results, while others question long-term efficacy and recurrence. This highlights the need for multi-center trials to clarify cryoablation’s role in breast cancer care, especially regarding survival and quality of life. Future research should identify patient-specific factors influencing cryoablation success, such as genetic markers and tumor characteristics, to develop personalized treatment plans. Incorporating cryoablation into comprehensive strategies could improve early-stage breast cancer management. Integrating cryoablation into clinical practice offers a non-invasive alternative for patients not suited for surgery, promoting a multidisciplinary approach for balanced treatment options. In conclusion, cryoablation shows promise for early-stage breast cancer, but further research is needed to refine its role in personalized treatment and improve patient outcomes.
